# The evolution of radical prostatectomy: a scoping review on surgical techniques and integration of surgeon-assisting concepts into the surgical workflow

**DOI:** 10.3389/fsurg.2026.1753503

**Published:** 2026-04-30

**Authors:** Philipp Nick, Theodoros Tokas, Selçuk Guven, Panagiotis Kallidonis, Eric Barret, Begoña Ballesta Martínez, Moaz F. Ismail Abdelrahman, Andrea Gallioli, Udo Nagele, Gernot Ortner

**Affiliations:** 1 Department of Urology and Andrology, General Hospital Hall I.T., Hall in Tirol, Austria; 2 Training and Research in Urological Surgery and Technology (T.R.U.S.T.)-Group, Hall in Tirol, Austria; 3 Department of Urology, University General Hospital of Heraklion, University of Crete, Medical School, Heraklion, Greece; 4 EAU Section of Endourology – Laparoscopy Working Group, Arnhem, The Netherlands; 5 Department of Urology, University of Konya, Konya, Türkiye; 6 Department of Urology, University of Patras, Patras, Greece; 7 Department of Urology, Institute Mutualiste Montsouris, Paris, France; 8 Department of Urology, University Hospital del Vinalopo, Elche, Spain; 9 Department of Urology, East Lancashire Hospitals NHS Trust, Blackburn, United Kingdom; 10 Department of Urology, Puigvert Foundation, Barcelona, Spain

**Keywords:** artificial intelligence, evolution, precision surgery, radical prostatectomy, robot-assisted surgery, single-port surgery

## Abstract

**Background:**

Radical prostatectomy remains a key curative treatment for localized prostate cancer which has undergone continuous transformation. Recent developments in robot-assisted and single-port surgery have followed the concept of minimizing morbidity while maintaining oncologic safety. Furthermore, the integration of artificial intelligence and novel diagnostic tools have transformed the modern surgical workflow. Therefore, the aim of this study is to give an overview of the evolution of surgical techniques, functional and oncological outcomes, and available surgeon-assisting concepts.

**Methods:**

We conducted a non-structured review to summarize the evolution of radical prostatectomy techniques and recent developments in surgeon-assisting tools. Relevant English-language publications were identified through a targeted PubMed search using predefined keywords related to, laparoscopic, robot-assisted, Retzius-sparing, and single-port approaches. Key studies, systematic reviews, and meta-analyses were used and synthesized to provide a comparative overview. Furthermore, studies focusing on integration of artificial intelligence, novel diagnostic tools and targeted surgery are discussed.

**Results:**

Laparoscopic surgery aims to reduce morbidity yet is technically demanding. Robotic-assisted approaches improve visualization and precision, leading to faster recovery and earlier continence recovery. Newer Retzius-sparing and single-port techniques show promising functional results, though evidence is still limited. Novel diagnostic tools including image overlay, targeted surgery, and fast and accurate intraoperative pathological assessment of resection margins are increasingly shaping modern radical prostatectomy.

**Conclusion:**

Radical prostatectomy has evolved into a minimally invasive, technology-driven procedure with improved recovery and functional outcomes. Advances in robotics, imaging, and artificial intelligence enhance surgical precision. Ongoing innovation and long-term data will define the future of prostate surgery.

## 1 Introduction

Radical prostatectomy remains one of the main curative treatment options for localized prostate cancer. Since the introduction of the anatomic radical retropubic approach by Walsh in the early 1980s, which emphasized the preservation of the neurovascular bundles, the procedure has continuously evolved (1). These anatomical findings formed the basis for modern prostate surgery and fundamentally changed the balance between cancer treatment and quality of life. Over time, continuous improvements in technique and technology have made radical prostatectomy a highly developed procedure that now includes open, laparoscopic, and robotic-assisted approaches (1–3). More recently, innovations such as Retzius-sparing and single-port robotic procedures, including transvesical and transperineal approaches, have expanded the boundaries of minimally invasive prostate surgery (4–7).

In this scoping review, we aim to provide a comprehensive overview of the historical development, technical evolution, and current evidence for the major surgical approaches to radical prostatectomy. By integrating data from key studies and systematic reviews, this work compares laparoscopic, robotic-assisted, and single-port methods with focus on perioperative parameters, functional recovery, and oncologic outcomes. In addition, recent advancements of integrating novel surgeon-assisting concepts into robot-assisted surgery and an outlook of future possibilities are discussed. Although individual surgical techniques and their outcomes have been reported extensively, overviews of the technological shift from open surgery to robot-assisted and single-port procedures and their collective impact on clinically relevant outcomes remain limited. This review therefore summarizes these developments and further describes current advances in surgeon assisting techniques to demonstrate how technological innovations are changing modern prostate cancer surgery.

## 2 Methods

A literature search was performed in November 2025 using the PubMed database to identify relevant studies published in English. The search strategy combined key terms such as “*radical prostatectomy”*, “*laparoscopic”*, “*robotic-assisted”*, “*Retzius-sparing”*, “*single-port”*, “*transvesical”*, “*transperineal”, “artificial intelligence”, “confocal”, “ICG”, and “precision surgery”*. Boolean operators and medical subject headings were used where possible to broaden the scope of the search.

Publications focusing on surgical techniques were screened by two reviewers (PN, GO) for relevance, extracted, and summarized, with a focus on perioperative parameters such as blood loss, hospital stay, recovery of continence, erectile function and oncological control. Furthermore, studies describing integration of artificial intelligence and precision surgery concepts were included if judged relevant. Selection of appropriate studies was performed via discussion of relevance to the review questions. Disagreement was resolved by a third independent reviewer (TT). Priority was given to recent systematic reviews, meta-analyses, and large single- or multi-center cohort studies. Primary descriptions of surgical approaches, such as the original reports by Walsh on open retropubic radical prostatectomy and by Binder and Kramer on the first robotic series, have also been included to provide historical and technical context (1, 2).

No formal quantitative analysis was performed, as the primary goal of this work was to provide a descriptive synthesis and qualitative comparison of established and emerging operative approaches. Accordingly, the manuscript was designed as a narrative overview rather than a systematic review intended to provide a clinically-focused comparison of all available evidence. The aim of the review is to summarize key developments in the field and present them in a structured manner for a broad clinical and scientific readership. Cross-references were manually reviewed separately by two of the researchers (PN, GO) to ensure that all important developments in surgical technique were represented. All included articles were analyzed in terms of their methodological quality and the relevance of their findings to current clinical practice.

## 3 Surgical approaches

### 3.1 Laparoscopic radical prostatectomy (LRP)

LRP was developed in the 1990s as a minimally invasive alternative to the open retropubic radical prostatectomy (RRP). Until then RRP which was popularized by Walsh in the 1980s was the only feasible surgical approach for treating localized prostate cancer (1, 3). LRP was pioneered by Schuessler et al. in 1991 and was subsequently refined by Guillonneau and Vallancien, whose standardization of the technique helped establish LRP as a feasible operation for localized prostate cancer (3, 8). The procedure is performed under general anesthesia, with the patient positioned in steep Trendelenburg to facilitate exposure of the pelvis. After trocar placement the prostate is approached either through a transperitoneal or an extraperitoneal route (8).

In the transperitoneal approach the abdominal cavity is usually accessed via the umbilicus, with the intestines being pushed cranially. The endopelvic fascia is opened, the dorsal venous complex is ligated, and the bladder neck is divided to access the prostate base. Dissection continues posteriorly to the seminal vesicles and vas deferens. The vesicourethral anastomosis is completed using continuous suturing (8). In contrast, the extraperitoneal approach avoids entering the abdominal cavity by creating a novel working space, typically using a balloon dissector to create a preperitoneal space (9). This technique minimizes the risk of intra-abdominal complications, such as postoperative ileus, and may be preferable in patients with prior extensive abdominal surgery, obese patients or patients with comorbidities affecting the peritoneal cavity. Several studies have demonstrated that both techniques yield similar operative times, positive surgical margin rates, and functional recovery, with the choice largely dependent on surgeon preference and patient anatomy (10). An overview of the advantages and disadvantages of the surgical techniques is presented in Table 1.

**Table 1:** Comparison of surgical approaches for radical prostatectomy.

Approach	Continence Recovery	Erectile Function	Blood loss	Hospital stay	Oncologic outcomes	Key remark
Open Retropubic	++	++^+^	−	−	+++	Reliable long-term data
Laparoscopic Transperitoneal	++	++	+	+	+++	Standard minimally invasive route
Laparoscopic Extraperitoneal	++	++	+	+	+++	Avoids intraperitoneal complications
Robotic Transperitoneal	++^+^	+++	++	++	+++	Standard robotic approach
Robotic Extraperitoneal	++^+^	+++	++	++	+++	Comparable outcomes with less bowel impact
Robotic Retzius-sparing	+++	+++	++	++	++	Fastest continence recovery
Single-port Transperitoneal	++^+^	+++	+++	+++	+++	Reduced incision trauma
Single-port Transvesical	+++	+++	+++	+++	+++	Early continence, no ePLND possible
Single-port Transperineal	+++	+++	+++	+++	+++	Ideal for patients with prior abdominal surgery, no ePLND possible

+++ = excellent, ++^+^ = very good, ++ = good, + = moderate, − = less favorable.

Compared with open surgery, LRP offers superior visualization, reduced blood loss, less postoperative pain, and shorter hospital stay. The magnified optical view allows for more precise dissection, especially around the apex and neurovascular bundles, which theoretically improves the potential for nerve sparing. Patients generally experience faster recovery, shorter catheterization time, and earlier return to normal activities. Oncologic control is comparable to open and robotic surgery when performed by experienced surgeons (11–13).

Despite these advantages, the laparoscopic approach has several limitations. The procedure is technically demanding and associated with a steep learning curve, requiring advanced laparoscopic skills and familiarity with pelvic anatomy. The operation also demands specialized equipment and often longer operative times compared to open surgery (11). In addition, the more demanding body posture required of the surgeon during LRP increases surgeon fatigue compared to robot-assisted procedures, which implies additional risks (14).

### 3.2 Multiport robotic-assisted laparoscopic radical prostatectomy (MP-RALP)

RALP has become the predominant surgical technique for localized prostate cancer in most oncological centers and represents the most significant advancement in minimally invasive urological surgery since the introduction of laparoscopy. Building upon the foundations of LRP, RALP utilizes robotic systems to overcome many of the technical limitations of conventional laparoscopy, providing enhanced three-dimensional visualization, articulated instruments that replicate the natural movements of the human wrist and a more relaxed working position. The first series of robot-assisted prostatectomies was reported by Binder and Kramer in 2001, using the da Vinci Surgical-System (InSite Vision Systems, Intuitive Surgical Inc., Mountain View, CA, USA) (2). Following the initial comparative study by Menon et al., RALP quickly gained worldwide attention and has since transformed prostate cancer surgery around the globe (15).

The procedure is similar to LRP performed under general anesthesia, with the patient positioned in steep Trendelenburg to facilitate exposure of the pelvis. After trocar placement, the prostate is approached again either through a transperitoneal or an extraperitoneal route with similar advantages and disadvantages as for LRP (2, 16).

A further refinement represents the Retzius-sparing approach, first described by Galfano et al. in 2010. This new technique accesses the prostate posteriorly through the pouch of Douglas, thereby preserving the anterior structures of the space of Retzius—including the puboprostatic ligaments, endopelvic fascia, and dorsal venous complex—that are traditionally divided during standard prostatectomy. By maintaining these anatomic structures, the Retzius-sparing technique aims to enhance early recovery of urinary continence (4). Several prospective studies and meta-analyses have confirmed superior early continence rates, often within days after catheter removal (17, 18). However, positive surgical margins in ≤ pT2 tumors seem to be statistically significantly higher than compared to traditional RALP approaches (17).

The key advantages of robotic-assisted surgery include considerably improved visibility, superior instrument flexibility, and greater precision, enabling careful preparation of the neurovascular bundles. These advantages result in less intraoperative blood loss, lower transfusion rates, shorter hospital stays, and faster recovery compared to open and conventional laparoscopic procedures. The functional outcomes, particularly with regard to erectile function, following RALP are favorable. The oncological outcomes are comparable to those of open or laparoscopic techniques (12, 13, 18, 19).

Furthermore, the literature suggests that the learning curve for RALP is also shorter than for conventional laparoscopy, allowing many surgeons to acquire the necessary skills more quickly. However, the learning curve itself varies greatly depending on various factors, such as previous surgical experience (20).

Nevertheless, robotic surgery is not without drawbacks. The most prominent limitation is the high cost associated with purchase and maintenance of the robotic system, as well as the disposable instruments, which may limit its accessibility. On the other hand, the shorter hospital stay associated with RALP rapidly reduces the overall costs of RALP compared to RPE, making it slightly financially more lucrative. Recent studies and meta-analyses suggest that RALP is indeed cost-effective compared to LRP in high-income countries (21). Moreover, RALP has proven to be more environmentally friendly than LRP, as it causes fewer CO2 emissions (22).

### 3.3 Single-port robotic-assisted laparoscopic radical prostatectomy (SP-RALP)

SP-RALP represents the latest refinement in the evolution of minimally invasive prostate surgery. Building upon the principles of multi-port robotic systems, single-port techniques aim to further reduce surgical morbidity by performing the entire operation through a single incision, usually through the lower abdomen (5, 23).

Similar to MP-RALP, the prostate can be accessed via a transperitoneal or extraperitoneal approach. The surgical steps are largely the same as those for MP-RALP, however instrument size and classical triangulation is reduced, and coordination of instruments is key. The flexible 360° endoscope provides good visibility even within the confined pelvic cavity (5). Studies suggest that single-port approaches achieve similar perioperative outcomes to standard multi-port RALP, but with less discomfort associated with the incisions (23, 24).

A novel adaptation of SP-RALP surgery is the transvesical approach, in which the prostate is accessed directly through the bladder. This technique represents a paradigm shift by entirely avoiding both the peritoneal cavity and the space of Retzius. After a small suprapubic incision, the single-port trocar is introduced into the bladder, which is then insufflated with carbon dioxide to create a working space. Dissection proceeds from inside the bladder neck towards the apex, and the prostate is mobilized under direct vision (6). By preserving the structures of the Retzius space, including the puboprostatic ligaments, the endopelvic fascia, and the dorsal venous complex, this approach, similar to Retzius-sparing RALP, aims to improve early recovery of continence. Early outcomes suggest that the transvesical SP-RALP can be safely performed with excellent early continence results, minimal postoperative pain, and same-day discharge in selected patients (18, 25). Another major advantage of the transvesical approach is that it can be performed under epidural anesthesia, eliminating the need for mechanical ventilation. In addition, there is no need for a steep Trendelenburg position, which is particularly beneficial for certain multimorbid patient groups (26). Nevertheless, this method is technically demanding, especially in patients who require extensive pelvic lymph node dissection, and may not be suitable for all cases (25).

More recently, the transperineal SP-RALP approach has gained attention as an alternative route that avoids both the peritoneal and bladder cavities. In this technique, a small perineal incision between the scrotum and the anus allows direct access to the prostate from below. The working space is created in the deep perineum, and the robotic system is docked in a caudal position relative to the patient. The transperineal route provides a short and direct path to the prostate, offering benefits in patients with prior major abdominal surgery, prior radiation, or in general hostile abdomens (7). Early comparative findings show promising results with low blood loss, short hospital stays, and continence rates comparable to those of conventional robot-assisted procedures, although large-scale data remains limited (7, 18).

Across all SP-RALP approaches, the reported advantages include reduced postoperative pain, smaller incisions, improved cosmetic results and potentially faster recovery, provided the surgeon is experienced in robotic single-port platforms, as the learning curve for this type of surgery is particularly steep (7, 23, 25, 27). However, these advantages are still rather theoretical. Further prospective studies are needed to compare and evaluate these new surgical approaches.

## 4 Discussion

### 4.1 Continence

Postoperative urinary continence remains one of the most important functional outcomes after radical prostatectomy. Recovery rates have improved substantially over the past decades, mainly due to enhanced understanding of the pelvic neuroanatomy first described by Walsh (1). Comparative analyses show that continence recovery in patients treated with RRP, LRP, and traditional RALP is generally similar (13, 18, 19). Nevertheless, more recent studies show an advantage for RALP, even at the beginning of the surgeons robotic learning curve (12, 28). The Retzius-sparing modification proposed by Galfano et al. further accelerates early continence by preserving the anterior supportive structures of the pelvic floor (4). Studies reported significantly higher immediate continence recovery compared with standard RALP (17, 18). However, long-term continence results beyond one year appear to be equivalent for all common robotic techniques, including the Retzius-sparing approach (18). Newer approaches such as the transvesical and the transperineal SP-RALP techniques, which also preserve the Retzius space, show promising initial continence results in recent studies. However, long-term data for these relatively new approaches are still lacking (7, 18, 24).

### 4.2 Erectile function and potency recovery

Erectile function after radical prostatectomy is a crucial factor for postoperative quality of life and is closely related to the extent of nerve preservation and surgical precision. In experienced hands RALP has shown improved results in terms of erectile function and restoration of potency when compared to RRP performed by the same surgeons (19). Further comparative analyses have demonstrated that RALP also achieves better functional results than LRP (12, 28). Recent studies on SP-RALP, particularly on the transvesical approach, show comparable or even improved functional outcomes compared to conventional multiport systems (24, 25). Overall, the transition from open and laparoscopic surgery to robotic techniques suggests to positively impact erectile function preservation after radical prostatectomy (12, 19).

### 4.3 Blood loss

Intraoperative blood loss and transfusion requirements have consistently declined with the evolution from open to minimally invasive and robotic procedures. RRP is usually associated with the highest blood loss, with up to 650 ml on average in experienced hands (19). The introduction of laparoscopy led to a substantial reduction in mean blood loss and transfusion rate (12). Robotic-assisted surgery further minimizes bleeding in some studies. Mean blood loss in RALP studies is commonly reported at an average of 200 ml, with transfusion rates at around 1.2% (12, 19). SP-RALP procedures show similarly low or even lower intraoperative blood loss (24). Overall, regardless of the approach, RALP offers the most consistent advantages in terms of hemostasis, with SP techniques showing the most promising results in recent studies (12, 19, 24).

### 4.4 Hospital stay and recovery

The length of hospital stay, and postoperative recovery vary considerably depending on the surgical approach and are closely related to the degree of surgical invasiveness. Patients undergoing RRP require longer inpatient treatment due to greater tissue trauma, more severe postoperative discomfort, and delayed mobilization (13, 19). The transition to laparoscopic techniques significantly improved perioperative recovery by reducing incision size, minimizing postoperative pain, facilitating earlier ambulation and shorter catheterization time (12). With the widespread use of robotic-assisted surgery, these advantages have become even more prominent. The reduced surgical trauma associated with RALP leads to shorter hospital stays, allowing patients to be discharged even on the same day in appropriate cases (12, 29). SP-RALP procedures represent a further development of this trend and demonstrate the potential for even faster recovery and minimal postoperative discomfort, as well as same-day discharge for carefully selected patients (23, 24).

### 4.5 Oncologic outcomes

The ultimate measure of the effectiveness of radical prostatectomy remains oncological control. Long-term data from open retropubic trials show excellent cancer-specific survival rates, setting a benchmark for newer techniques (11, 19). Several studies have confirmed that laparoscopic and robot-assisted procedures performed by experienced surgeons lead to equivalent oncological outcomes in terms of positive surgical margins and biochemical recurrence rates (11, 12, 19). Some more recent studies even suggest an advantage in favor of RALP in terms of oncological control (12, 30). The Retzius-sparing technique shows to have a slightly higher positive margin rate for tumors ≤ pT2 compared to standard RALP (17). Single-port procedures have so far shown similar positive surgical margin rates compared to MP-RALP, although oncological long-term follow-up is still lacking (23, 24). Despite recent evidence suggests an improved cancer specific mortality following RALP vs. RRP (30), no collective oncologic superiority can be generally reported for any operative approach; surgical expertise and patient selection continue to outweigh the influence of access route or technology (12, 19, 23).

### 4.6 Surgeon-assisting concepts

#### 4.6.1 AI- and AR-powered intraoperative support

Artificial intelligence (AI) has become a key driver of innovation in radical prostatectomy, particularly in the field of robot-assisted surgery. The integration of machine learning and computer vision into robotic systems offers new opportunities to improve intraoperative precision, increase perioperative efficiency, and individualize treatment through real-time data analysis and decision support (31). In particular, AI-based systems can be extremely useful in intraoperative tasks such as detection of anatomical landmarks, prediction of adverse events, and real-time decision support (32–34). These applications address critical steps in radical prostatectomy, where the surgeon must balance oncological radicality with the preservation of functional structures.

Augmented reality (AR) and three-dimensional (3D) visualization techniques enable intraoperative projection of patient-specific anatomical models directly onto the surgical field, providing surgeons with a real-time digital map of tumor margins and surrounding neurovascular structures. This approach helps precise dissection, increasing both safety and precision. Evidence from the randomized RIDERS trial showed that 3D—AI assisted—AR guidance during RALP significantly reduced positive surgical margins and improved early continence outcomes compared to conventional MRI-based navigation (32). Despite these promising results, AR-assisted surgery still faces important technical challenges. Current systems often require specialized personnel and considerable manual preparation to align the virtual model with the real surgical field. During the operation, accurate image matching is only possible under favorable conditions, such as the presence of a urinary catheter and a clear and largely blood-free resection field. Therefore, reliable matching between virtual and real anatomy remains difficult and continues to limit routine clinical use (32).

Beyond navigation, AI-based systems are being developed to support real-time intraoperative safety. A recent example is the Bleeding Artificial Intelligence Detector (BLAIR) system capable of detecting and forecasting bleeding events during RALP, outperforming human recognition in early phases of bleeding onset. Therefore, BLAIR can help the surgeon to respond proactively before bleeding becomes clinically relevant (33). Overall, these technologies are most likely to provide clinical benefit when they assist high-risk or high-precision steps of the procedure. However, many of these systems remain in early developmental stages, and their widespread adoption will depend on improved technical robustness, seamless integration into surgical workflows, and demonstration of clear clinical benefit in prospective studies.

#### 4.6.2 Intraoperative margin assessment

The NeuroSAFE technique represents a major step in improving intraoperative margin control during nerve-sparing RALP. It involves a systematic frozen-section analysis immediately after the prostate is removed. The surgeon temporarily halts the procedure while a pathologist examines circumferential frozen sections to detect possible positive margins. If malignant tissue is identified, the corresponding site can be partially or fully resected during the same operation, ensuring complete oncologic clearance while preserving the residual nerval fibers whenever possible (35). The NeuroSAFE PROOF trial, a multicenter randomized phase 3 study, demonstrated that this real-time pathological assessment significantly improves postoperative erectile function and early urinary continence compared with standard RALP, without compromising oncologic safety (35). Building on this concept, fluorescence confocal microscopy (FCM) offers a rapid, optical method for intra operative margin assessment. Implemented in the LaserSAFE technique, FCM generates high-resolution digital scans of the prostate surface, avoiding the time and tissue preparation required for frozen sections. Early studies have shown good correlation with final histopathology. In addition, the digitalized scans offer the potential for AI-supported image interpretation, which could automate the margin assessment and further accelerate intraoperative decision-making (36).

#### 4.6.3 PSMA- and fluorescence-guided surgery

A novel technique to enhance the accuracy of extended pelvic lymph-node dissection (ePLND) is the prostate-specific membrane antigen (PSMA)–radioguided surgery. In this approach, a PSMA-targeting tracer labeled with a gamma-emitting radionuclide is injected intravenously before surgery. The tracer selectively binds to PSMA-expressing metastatic tissue. During the operation, a gamma probe integrated into the robotic system detects signals from affected lymph nodes in real time, guiding the surgeon toward metastatic tissue. This method ensures that small or atypically located metastases are not missed, while simultaneously reducing unnecessary dissection of healthy tissue (37). In parallel, indocyanine-green (ICG) fluorescence imaging offers another visualization strategy. After prostatic injection of the ICG dye, fluorescence optics on the robotic platform illuminate the lymphatic pathways draining the prostate, allowing identification of sentinel and regional nodes during ePLND (38). Intraoperative 3D-AR can also assist surgeons during PLND by overlaying a patient-specific 3D model, created from a preoperative PSMA PET scan, over the real-time robotic endoscopic view. This enables precise intraoperative localization of PSMA-positive nodes (34).

### 4.7 Telesurgery and future directions

Another advantage of robotic systems is the possibility of telesurgery, which allows surgeons to perform operations from a remote location by remotely controlling robotic instruments via secure high-speed communication networks. This technology has the potential to reduce geographical barriers in access to highly specialized surgical care and may be particularly relevant for underserved or resource-limited regions. Recent studies have demonstrated its growing feasibility: Patel et al. successfully performed a trans-Pacific telesurgery between Orlando and Shanghai in animal models using 5G and fiber-optic connections (39). Aldousari et al. reported a human RALP conducted remotely between Shanghai and Kuwait with minimal latency and favorable outcomes (40). Recently published outcomes by Moschovas et al. on two successful RALPs performed via telesurgery in Angola, demonstrate the feasibility and potential of offering highly specialized surgeries in resource-poor regions (41). Recently, the first European telesurgical prostatectomy was performed successfully by Prokar Dasgupta between London (UK) and Gibraltar (42). However, despite these encouraging reports, telesurgery remains in an early phase of clinical adoption and still depends on highly stable network infrastructure, cybersecurity, local support teams, and careful patient selection. In 2025, the Live Surgery Committee of the European Association of Urology developed guidelines for the widespread implementation of safe telesurgery (43).

A major future prospect is the approach of robot-assisted autonomous surgery. Successful cholecystectomies have already been performed on *ex vivo* models, carried out exclusively by a robot without human intervention (44). This holds enormous potential to automatically and autonomously perform at least routine and standardized steps in urological surgery, such as bladder neck dissection during RALP, in the near future. As telecommunication infrastructure advances and more robotic platforms become available, telesurgery and autonomous surgery may enable access to specialized surgery worldwide in the future (41, 44).

Another promising aspect of robotic surgery is teleproctoring, which enables remote monitoring by experts during training, certification, and complex live procedures. This approach can facilitate structured skills transfer, speed up the introduction of new robotic techniques, and expand access to experienced mentors, especially for surgeons and centers that do not have immediate expert support on site. Available evidence suggests that teleproctoring is technically feasible and can support the spread of robotic expertise without risking perioperative safety or short-term surgical outcomes. Furthermore, it can help break down geographic barriers, improve training efficiency, and reduce the logistical burden associated with in-person supervision. However, its broader implementation depends on a reliable telecommunications infrastructure, data security, and clarification of legal responsibilities (45).

In addition, although robotic surgery is associated with high acquisition and maintenance costs, RALP may become cost-effective compared to LRP in high-volume centers because fixed platform costs can be distributed across larger caseloads, while shorter hospital stay, faster recovery, reduced complication burden, and more efficient perioperative workflows may improve the overall economic balance (21). Furthermore, the recent introduction of multiple novel robotic platforms on the market has led to competition on the market which will lead to lower acquisition and maintenance costs (46, 47).

Due to the increasingly high-tech and data-driven nature of modern surgery, the successful implementation of these innovations will depend heavily on interprofessional collaborations. Furthermore, the next stage of development for AI-based tools requires large, standardized datasets and international collaborations such as the PIONEER project to build high-quality big data infrastructures for urological research (48). At the same time, it must be recognized that for most new AI-driven systems, AR platforms, and surgical assistance tools, there are currently no long-term clinical outcomes data available, making their advantages over conventional techniques uncertain.

### 4.8 Limitations

This review has several limitations. First, it represents a qualitative narrative overview rather than a formal systematic review or meta-analysis, and therefore the literature selection may be subject to selection bias. Second, the available studies on radical prostatectomy are heterogeneous, with considerable variation in definitions of key outcomes such as urinary continence, erectile function recovery, and perioperative parameters, which limits direct comparability between surgical approaches. Third, many emerging techniques—particularly single-port procedures and novel surgeon-assisting technologies—are supported primarily by early clinical studies with limited sample sizes and relatively short follow-up. Despite these limitations, this manuscript provides a structured review of the technological evolution of radical prostatectomy.

## 5 Conclusions

Over the past decades, radical prostatectomy has evolved from an open procedure into a highly minimally invasive operation driven by robotic and digital innovations. Recent evidence confirms that robotic-assisted approaches achieve equivalent oncologic outcomes while improving perioperative recovery, continence, and potency preservation. Among these developments, MP-RALP is now sufficiently established for broad clinical adoption, given its mature evidence base. In contrast, newer strategies such as SP-RALP, AR guidance, AI-based intraoperative decision support and PSMA-guided surgery remain highly promising by enhancing precision and functional results, but still require further validation before routine implementation. The future of these techniques in clinical practice will depend not only on technical feasibility, but also on practical factors such as acquisition costs, institutional resources, the learning curve for surgeons, and equal access for both high-volume centers and less specialized hospitals. Telesurgical connectivity, on the other hand, shows the potential for global access to expert care. The arrival of new robotic systems on the market will lead to competition and a reduction in the price of robotic surgery, making it available to a wider range of patients worldwide in the near future. Future research should focus on long-term comparative studies with standardized outcome definitions, including direct evaluation of AI-assisted vs. conventional robotic surgery. Continuous technological advances, combined with long-term data, will determine the impact of these innovations on the future of prostate cancer surgery.
